# 

**Published:** 1910-11-05

**Authors:** 


### CONTENTS.

| | PAGE |
|---|---|
| **The Plague in Suffolk** ... | 153 |
| **The Founder of the Red Cross Association** ... | 154 |
| **Annotations**—An Echo of the War—Prevention of Abortion in Belgium—An Unsatisfactory Appointment ... | 155 |
| **Medical Opinion and Movement** ... | 156 |
| **Hospital Clinics**—The Girdle-test (l'Epreuve de la Sangle). By A. Chaillou, M.D., and Leon MacAuliffe, M.D.... | 159 |
| **Practical Notes on Diagnosis and Treatment** ... | 162 |
| **Surgery**—Chronic Gonorrhœa in the Female ... | 163 |
| **Medico-Legal Points**—Workmen's Compensation Cases ... | 164 |
| **Medical Antiquities**—The Maxims of John Arderne, Master Surgeon ... | 165 |
| **Tropical Medicine**—Bilharziosis in Girls and Women ... | 166 |
| **News and Events of the Week** ... | 167 |
| **Appointments and Resignations** ... | 168 |
| **Obituary** ... | 168 |
| **The Week's Work**—King Edward Memorials—Sheffield Royal Hospital—Hospitals and the Coronation—The Late Henri Dunant—Hospital Sunday in Hull—A Case for a Coroner—This Week's Drug Market ... | 169 |
| **Special Institutional Articles**—Eminent Chairman Series. II.—Sir Edward Wood ... | 171 |
| Institutional Treatment of Tuberculosis ... | 175 |
| The Late Prince Francis of Teck and the Middlesex Hospital . | 178 |
| **The Hospital World** ... | 179 |
| **Editor's Letter-Box**—The Plague ... | 180 |
| **Institutional Notes and News**... | 181 |
| **Gifts and Bequests** ... | 182 |
| **Jottings** ... | 183 |
| **New Appliances and Things Medical** ... | 184 |

